# Mendelian randomization studies of brain MRI yield insights into the pathogenesis of neuropsychiatric disorders

**DOI:** 10.1186/s12864-021-07661-8

**Published:** 2021-06-02

**Authors:** Weichen Song, Wei Qian, Weidi Wang, Shunying Yu, Guan Ning Lin

**Affiliations:** 1 Shanghai Mental Health Center, School of Biomedical Engineering, Shanghai Jiao Tong University School of Medicine, Shanghai Jiao Tong University, 200030 Shanghai, China; 2 Shanghai Key Laboratory of Psychotic Disorders, 200030 Shanghai, China

**Keywords:** Neuroimaging, Neuropsychiatric disorders, Dysconnectivity, Anorexia nervosa, Superior longitudinal fasciculus

## Abstract

**Background:**

Observational studies have identified various associations between neuroimaging alterations and neuropsychiatric disorders. However, whether such associations could truly reflect causal relations remains still unknown.

**Results:**

Here, we leveraged genome-wide association studies (GWAS) summary statistics for (1) 11 psychiatric disorders (sample sizes varied from *n* = 9,725 to 1,331,010); (2) 110 diffusion tensor imaging (DTI) measurement (sample size *n* = 17,706); (3) 101 region-of-interest (ROI) volumes, and investigate the causal relationship between brain structures and neuropsychiatric disorders by two-sample Mendelian randomization. Among all DTI-Disorder combinations, we observed a significant causal association between the superior longitudinal fasciculus (SLF) and the risk of Anorexia nervosa (AN) (Odds Ratio [OR] = 0.62, 95 % confidence interval: 0.50 ~ 0.76, *P* = 6.4 × 10^− 6^). Similar significant associations were also observed between the body of the corpus callosum (fractional anisotropy) and Alzheimer’s disease (OR = 1.07, 95 % CI: 1.03 ~ 1.11, *P* = 4.1 × 10^− 5^). By combining all observations, we found that the overall *p*-value for DTI − Disorder associations was significantly elevated compared to the null distribution (Kolmogorov-Smirnov *P* = 0.009, inflation factor λ = 1.37), especially for DTI − Bipolar disorder (BP) (λ = 2.64) and DTI − AN (λ = 1.82). In contrast, for ROI-Disorder combinations, we only found a significant association between the brain region of pars triangularis and Schizophrenia (OR = 0.48, 95 % CI: 0.34 ~ 0.69, *P* = 5.9 × 10^− 5^) and no overall p-value elevation for ROI-Disorder analysis compared to the null expectation.

**Conclusions:**

As a whole, we show that SLF degeneration may be a risk factor for AN, while DTI variations could be causally related to some neuropsychiatric disorders, such as BP and AN. Also, the white matter structure might have a larger impact on neuropsychiatric disorders than subregion volumes.

**Supplementary Information:**

The online version contains supplementary material available at 10.1186/s12864-021-07661-8.

## Background

Neuroimaging study is the most widely used procedure for studying brain disorders [1]. Many neuroimaging studies in the past quarter-century have revealed brain abnormalities in neuropsychiatric disorders [1, 2], which have served as the basis for biomarker discovery, clinical guidance, and investigations into the mechanisms of neuropsychiatric disorders [2, 3]. However, it is unclear whether such associations reflect disease causality [4]. One concern is that the spurious correlations [5] could emerge from indirect correlations with confounders such as medication, circadian and dietary changes, or false-positive events. Another issue is the direction of causality: the neurotoxicity hypothesis [6] suggests that psychiatric illnesses have toxic effects on the central nervous system, which leads to structural alterations following disease onset [7]. This theory gained support from several observations in which neuroimaging abnormalities exhibited dynamic progression during the neuropsychiatric disorder course [6, 7]. Under such theories, these associations should be utilized as clinical biomarkers rather than mechanism identifiers.

Several case-control studies with large sample sizes [8, 9] have found a significant correlation between neuroimaging alteration and neuropsychiatric disorder, yet still unable to distinguish the causality from the correlation. Longitudinal analyses have partially overcome the limitations of cross-sectional observational studies [10] for investigating the disease causality, such as detecting neuroimaging alterations in participants with the so-called high-risk status along with their progression [11, 12]. However, these studies are still limited by our current definition of high-risk cohorts. For example, the onset of neuropsychiatric disorders may be far earlier than a clinically recognizable high-risk status, such that the associated abnormalities may include changes that occurred after the primary pathology.

**Fig. 1:**
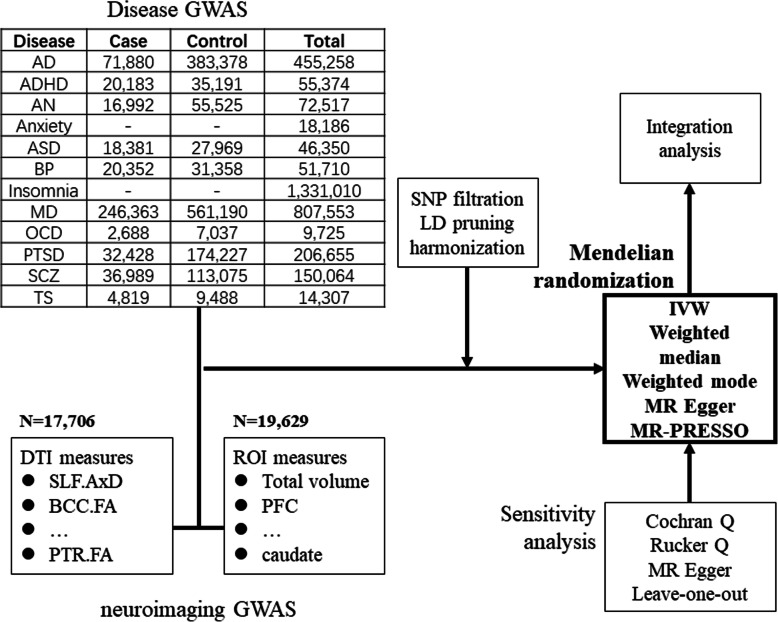
Flowchart of the study. Integrative analysis: *p* value distribution of MR result

Despite the longitudinal analysis focusing on the high-risk status, an alternative method for addressing the challenge of causality is Mendelian Randomization (MR) [13]. MR has been used to derive the relationships between peripheral inflammatory markers and schizophrenia [14] and between physical activity and depression [15] with great successes. By selecting genetic locus that is strongly associated with exposure, so-called instruments, MR separates subjects into high and low lifetime exposure groups according to their genotypes on the instruments [16], then compares the prevalence of outcomes between the high and low exposure groups. Such grouping is considered unbiased as genotypes are randomly determined during meiosis. Furthermore, a two-sample Mendelian Randomization (MR) estimates instrument-exposure and instrument–outcome relationships in different cohorts to infer the exposure-outcome relationship, without the need for information on individuals [16]. The explosive growth of genome-wide association studies (GWAS) offers opportunities for applying MR to solve the debate of causality in different clinical medicine fields. Recently, two GWAS from UK-Biobank (UKBB) [17, 18] revealed the genetic basis of brain structural measurements, providing an opportunity to address clinical neuroimaging studies’ causality. In the present study, with the summary-statistics GWAS data of Magnetic resonance imaging (MRI) (*N* = 17,706 and 19,629) and twelve neuropsychiatric disorders (*N* = 9,725 to 1,331,010), we implemented a two-sample MR approach to detect the causal relationship between white matter (WM) structures (diffusion tensor imaging [DTI]), brain subregion volumes (region of interest [ROI]), and neuropsychiatric disorders. An overview of the study design is illustrated in Fig. 1.

## Results

### Causal associations between DTI and neuropsychiatric Disorder

**Fig. 2:**
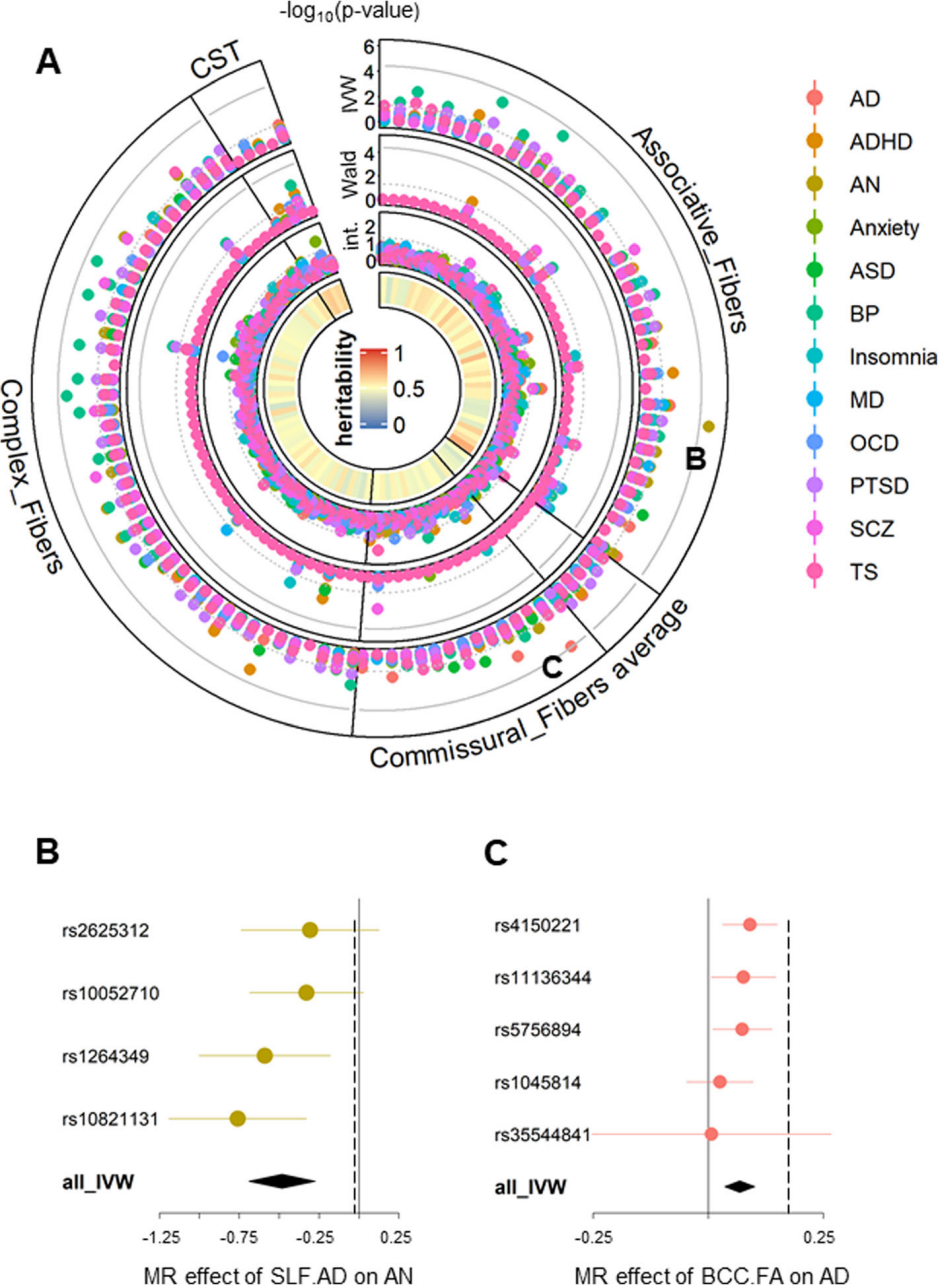
MR results for DTI-Disease associations. **a**: Each radius represents one DTI measures (110 in total: 22 white matter tracts × 5 DTI parameters). From outer to inner layer: -log_10_(p) for MR-IVW; -log_10_(p) for MR-Wald Ratio; -log_10_(p) for intercepts (int.) of MR-Egger regression; heritability of each DTI measures. The dotted grey line indicated the nominal p threshold (0.05); solid grey line indicated study-wide significance p threshold (0.05/1253). CST: Corticospinal tract. **b&c**: Forest plots showing MR effect of Superior longitudinal fasciculus (axial diusivities) on Anorexia nervosa (SLF.AxD-AN) and body of corpus callosum (fractional anisotropy) on Alzheimer Disease (BDD.FA-AD). Each line showed the single SNP MR effects (95 % confidence interval) estimated by Wald Ratio, and the last line showed the meta-analysis results calculated by IVW. Vertical dashed line indicated the Egger estimation of MR effect

After data harmonization, 1253/1320 (110*12) DTI-Disorder pairs and 973/1212 (101*12) ROI-Disorder pairs had at least one strong instrument (SNP with *P* < 5 × 10^− 8^ in DTI GWAS [17], Additional file 1) and were analyzed by MR (Additional file 2 and 3). We started by evaluating the DTI-Disorder association. In the inverse variance–weighted (IVW) analysis (outer layer of Fig. 2a), which required at least two instruments, one DTI-Disorder pair achieved study-wide significance (SW, *P* < 0.05/1320): superior longitudinal fasciculus Axial Diffusivity (SLF.AxD) – the risk of Anorexia nervosa (AN) (Fig. 2b; Table 1, odds ratio [OR] = 0.62, 95 % confidence interval [CI], 0.50 to 0.76, *P* = 6.4 × 10^− 6^). Another DTI-Disorder pair, body of corpus callosum Fractional Anisotropy (BCC.FA) − Alzheimer disease (AD), was close to reaching SW significance (Fig. 1c and Table 1, OR = 1.07, 95 % CI, 1.03 to 1.11, *P* = 4.1 × 10^− 5^). Both associations had relatively consistent results across instruments (Fig. 2b, c), in accordance with the fact that no outlier was detected (Additional file 2). Five other DTI-Disorder pairs also reached single-disease significance (*P* < 0.05/110) (Table 1). No significant result was found for the Wald ratio (second layer of Fig. 1a) applied to DTI-Disorder pairs with only one instrument.

**Table 1:** All DTI-Disorder pairs reaching Single Disease significant threshold (*p* < 0.05 after correction)

disease	DTI	IVW	Egger	Wtd.median	CochranQ	intercept
AN	SLF.AxD	-0.48(6.4 × 10^− 6^)	-0.02(0.97)	-0.41(0.002)	3.22(0.36)	-0.04(0.52)
AD	BCC.FA	0.07(4.1 × 10^− 5^)	0.18(0.31)	0.08(3.6 × 10^− 4^)	2.20(0.70)	-0.007(0.50)
BP	PTR.FA	-0.31(0.0002)	-0.22(0.53)	-0.27(0.008)	2.70(0.44)	-0.010(0.76)
BP	FX.FA	-0.31(0.0003)	0.60(0.30)	-0.27(0.01)	6.60(0.47)	-0.06(0.13)
BP	CGH.MD	0.31(0.0003)	0.11(0.70)	0.29(0.004)	3.13(0.37)	0.02(0.48)
BP	RLIC.RD	0.26(0.0004)	0.11(0.67)	0.22(0.01)	6.92(0.33)	0.01(0.55)
ADHD	ALIC.FA	-0.44(0.0004)	-1.33(0.41)	-0.48(0.001)	2.62(0.27)	0.08(0.53)

Results were shown as estimate (*P* value). *IVW* Inverse Variance Weighted sum of Wald Ratio, *Wtd.median* MR effects estimated by weighted median, *Intercept *intercept of egger regression, *intercept* Egger intercept. The definition of DTI acronym are provided in the Method section

A basic assumption of MR is that MR genetic instruments should only impact the outcome via the exposure and not any other pathway (horizontal pleiotropy) [16]. The existence of heterogeneity, which is introduced by outlier instruments, can also bias the MR estimation [19]. By applying various sensitivity tests, we confirmed that our results were not impacted by horizontal pleiotropy (Egger intercept *P* > 0.05; the third layer of Fig. 1a and Additional file 2) or heterogeneity (modified Cochran’s Q test *P* > 0.05; Table 1), or bidirectional effects (reverse MR *P* > 0.05; Additional file 2). They also showed consistent trends across different MR methods, which are robust against pleiotropy and measurement error (see Method for detail) (except for Fornix.FA − bipolar disorder [BP]; Table 1). They also showed little directional pleiotropy, as indicated by funnel plots (Additional file 4). Neither MR-PRESSO nor leave-one-out tests found the impact of outliers on these DTI-Disorder pairs (Additional files 2 and 4). Taken together, these results confirmed the casual relation between SLF.AxD-AN, and suggested potential relations of other five DTI-Disorder pairs.

### Overall contribution of DTI on neuropsychiatric disorders

**Fig. 3:**
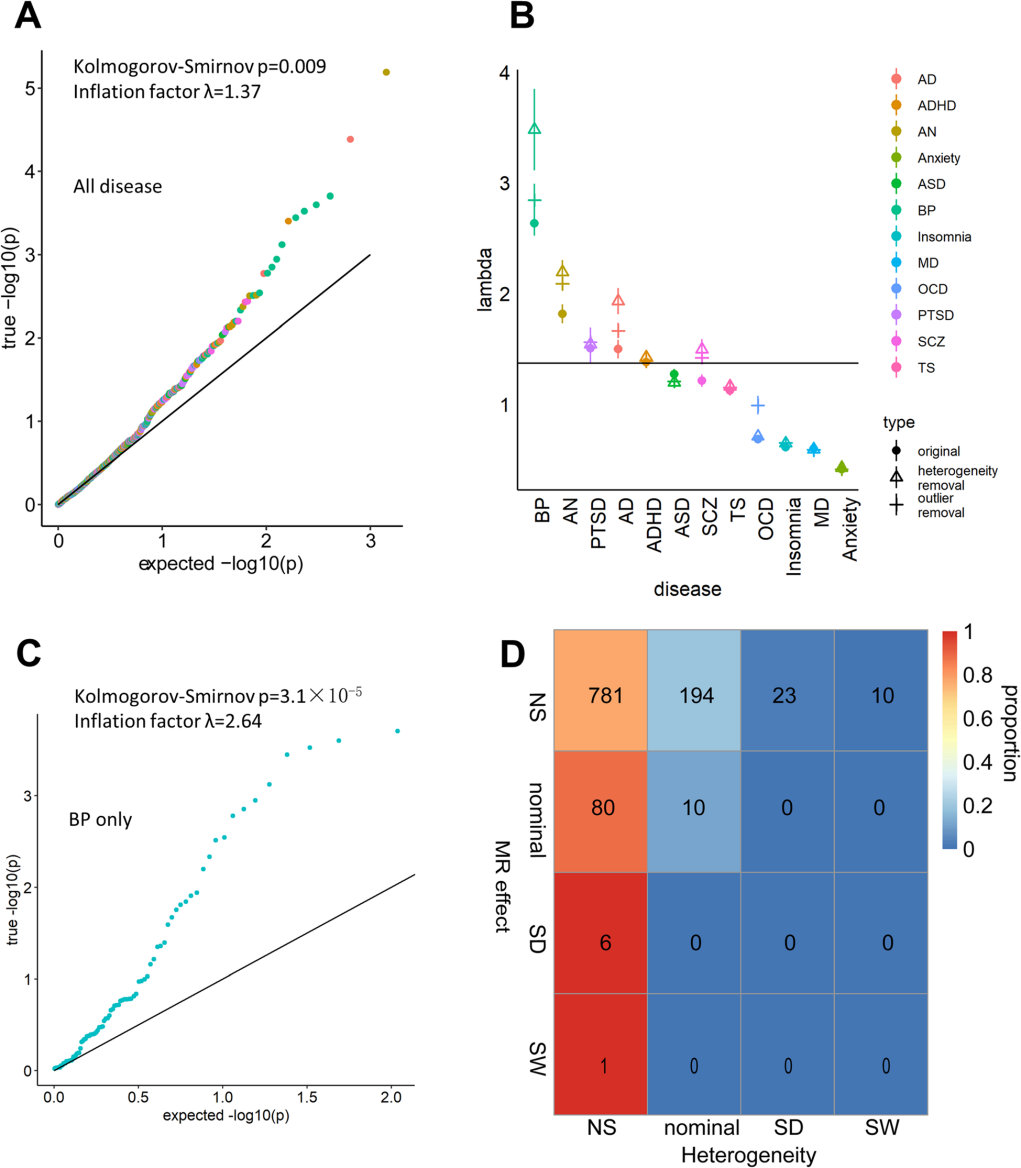
General contribution of DTI polymorphism to neuropsychiatric disorders.** a**: Quantile-Quantile (QQ) plot showing the distribution of all MR p values for the DTI-Disease (DD) association. **b**: Disease-specific inflation factor (λ). Solid points showed λ with no adjustment and were corresponded to λ in Table 2. Triangular points showed λ after removal of all DTI-Disorder pairs with significant heterogeneity. Cross points showed λ of p values which were calculated after removing all outlier SNPs (detected by MR-PRESSO) for each DTI-Disorder pair. Vertical error bars indicated a 95 % confidence interval. **c**: QQ plot for DTI-BP associations. **d**: Rank-Rank overlaps between MR effect and heterogeneity. The color of each grid corresponded to the proportion of each MR effect rank (each row summed up to 1). The number in each grid showed the exact number of DTI-Disorder pairs. NS: non-significant. Nominal: *p* < 0.05. SD: Single Disease level significance, *p* < 0.05/110. SW: Study-Wide significance, *p* < 0.05/1253

Despite separate DTI-Disorder pairs that reached the significance threshold, we were also interested in whether DTI as a whole made a causal contribution to the diseases. To answer this question, we pooled the IVW *P* value for all DTI-Disorder pairs and compared them to the null uniform distribution (Fig. 3a and Additional file 2). The distribution of IVW *P* was significantly inflated (KS *P* = 0.009, λ = 1.37); this result persisted after removing heterogeneous DTI-Disorder pairs (those with Cochran’s *P* < 0.05) (KS *P* = 0.002, λ = 1.48) or outlier instruments (SNPs with MR-PRESSO *P* < 0.05) (KS *P* = 8.0 × 10^− 5^, λ = 1.49). When we analyzed each disease separately (“Original” in Fig. 3b), BP (Fig. 3c) and AN showed the most significant inflation (Table 2). These results were also relatively stable against heterogeneity and outlier removal (Fig. 3b and Additional file 4). The permutation test confirmed that the results for BP (permutation *P* value [pp] for KS test: 0.012; pp for λ: 0.002) and AN (pp for λ: 0.012) were not due to the bias inherent in the data or method. Additionally, heterogeneous DTI-Disorder pairs generally had non-significant MR results and did not contribute to inflation (Fig. 3d and Additional file 2). Thus, we concluded that DTI polymorphisms made an overall causal contribution to neuropsychiatric disorders, especially BP and AN.

**Table 2:** Disease-specific *p* value distribution

disease	Subregion volume (ROI)		White matter structure (DTI)	
	lambda	p_ks	lambda	p_ks
AD	1.15(1.07~1.23)	0.92	1.50(1.42~1.58)	0.57
ADHD	1.79(1.74~1.86)	0.21	1.38(1.35~1.41)	0.13
AN	1.20(1.12~1.30)	0.72	1.82(1.73~1.90)	0.14
anxiety	1.04(1.01~1.09)	0.35	0.41(0.40~0.43)	0.0090
ASD	1.08(1.05~1.11)	0.36	1.27(1.23~1.32)	0.021
BP	1.10(1.07~1.15)	0.49	2.64(2.52~2.75)	3.1×10^-5^
Insomnia	1.42(1.38~1.47)	0.29	0.61(0.59~0.63)	0.061
MD	1.10(1.05~1.16)	0.49	0.60(0.58~0.62)	6.6×10^-4^
OCD	0.95(0.91~0.99)	0.09	0.68(0.67~0.70)	0.15
PTSD	0.81(0.75~0.89)	0.49	1.51(1.38~1.63)	7.8×10^-8^
SCZ	1.94(1.90~2)	0.0011	1.21(1.16~1.27)	0.0075
TS	1.02(1.00~1.05)	0.78	1.12(1.08~1.17)	9.3×10^-4^

Inflation factor (lambda) shown as estimate (95 % confidence interval). p_ks: *p* value for Komogorov-Smirov test for uniform distribution

### Causal associations between brain volume and neuropsychiatric Disorders

**Fig. 4:**
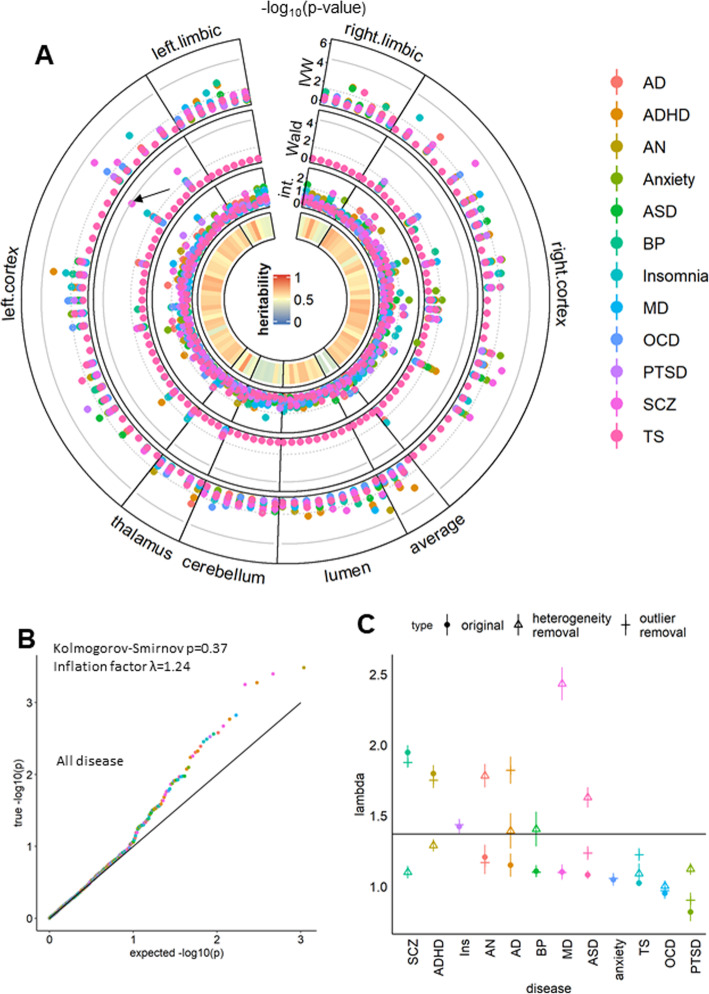
MR results for ROI-Disease associations. **a**: Circos plot for ROI-Disease (RD) relations, similar to Fig. 1a. Arrow indicated result for pars triangularis-schizophrenia association, which was described in detail in the main text. **b**: QQ plot for all IVW p values of RD pairs, similar to Fig. 2a. **c**: Disease-specific inflation factor (λ), similar to Fig. 2b

Similar to the DTI-Disorder analysis, we also assessed the ROI-Disorder association. 973/1212 (101*12) RD pairs had at least one strong instrument (SNP with *P* < 5 × 10^− 8^) and were analyzed by MR. In the IVW analysis, we found no SW significant results (outer layer of Fig. 3a and Additional file 3). The Wald ratio revealed a marginal SW result for pars triangularis (PT)-SCZ (OR = 0.48, *P* = 5.93 × 10^− 5^) (second layer of Fig. 4a), which was driven by a single SNP (rs2279829). The only trait associated with rs2279829 in PhenoScanner [20] was daytime dozing or sleeping (*P* = 2.02 × 10^− 6^). The SMR-HEIDI test detected a significant MR effect in the same direction (OR = 0.48, *P* = 7.57 × 10^− 4^) with no evidence of colocalization pleiotropy (HEIDI *P* = 0.84). We estimated that per 1-SD increment in normalized PT volume, the risk of SCZ decreased by 52 % (OR = 0.48). In conclusion, these results suggested a potential causal relationship between PT and SCZ. However, the validation of this relationship requires more work on potential pleiotropy.

The overall distribution of the IVW p-value did not significantly differ from the null distribution (Fig. 4b), even after removing heterogeneity (KS *P* = 0.12) and outliers (KS *P* = 0.07). However, IVW p-value for SCZ showed significant inflation (λ = 1.90, KS *P* = 0.001), and the result was significantly impacted by heterogeneity (Fig. 4c). Additionally, 10 % (6/58) of the nominally significant RD pairs showed heterogeneity, and 3 contained outlier SNPs (Additional files 3 and 4). In conclusion, there was no evidence that ROI polymorphisms had a universal contribution to neuropsychiatric disorders.

## Discussion

The issue of causality has long beleaguered clinical neuroimaging studies [4]. Confirmation of a causal change can provide insights into disease mechanisms at the circuit and region levels and may reveal useful biomarkers for predicting prognosis. Many current neuroimaging studies cannot directly draw this conclusion [1, 4], which partly limits the translation of their findings to clinical practice. In this study, we conducted hypothesis-free, data-driven MR analyses to assess the causal relationship between neuroimaging polymorphisms and neuropsychiatric disorders in an unbiased manner.

Our results showed that, in general, WM connectivity was more closely associated with the risk of neuropsychiatric disorders than gray matter volume (GMV) (Fig. 3a and 4b). Among neuropsychiatric disorders, BP showed the most significant association with genetically determined connectivity polymorphisms (Fig. 3b,c). These results support the dysconnectivity theory [21] of psychiatry, positing that major psychiatric disorders such as SCZ and BP have common WM abnormalities in their pathology [22]. According to this hypothesis, anatomic and neurodevelopmental changes that arise from neurotoxicity [6] are a consequence rather than a cause of the illness. Indirect evidence from a functional study of neuromodulation and myelination [21] and a case-control study of the high-risk state [12] supports the dysconnectivity theory. Research interest has now shifted from the region-of-origin to a connectome’s concept [22], in which connections between brain regions rather than the regions themselves cause BP and other mental illnesses. Our finding that DTI is more closely associated with the onset of neuropsychiatric disorders than ROI provides supportive evidence for this paradigm shift.

Our study’s top MR result was a novel risk factor for AN—namely, decreased SLF.AxD. A few studies have reported a decreased SLF integrity in AN patients [23, 24], but the results were inconsistent with the marginal effect size. As neuroimaging findings in AN patients are influenced by dietary and metabolic alterations [25], a causal change may not be manifested as a visible signal. Nonetheless, the SLF may contribute to body image distortion in the pathology of AN [24], probably through its connection to areas responsible for body image perception (prefrontal and parietal networks) and self-perception (inferior parietal lobe) [26]. Although abnormalities in both GMV [26] and WM connectivity [23, 24] have been observed, our results suggest that the latter is a primary cause. In contrast, the former is a consequence of neuromodulator mechanisms such as activity-dependent pruning [21].

The roles of BCC in AD and PT in SCZ—2 marginally significant results from our MR analysis—have received more attention in the literature than SLF in AN. However, both MR results should be interpreted with caution. BCC atrophy is widely observed in AD patients even at an early stage and reflects Wallerian degeneration and myelin breakdown [27]. However, our MR analysis revealed a reverse association: the FA of BCC was positively associated with AD risk (β = 0.07). One possible explanation for this discrepancy is that an enlarged BCC in early life is a risk factor for AD development at an older age, causing BCC atrophy after disease onset. Confirmation of such a complex theory requires more robust evidence from large-scale longitudinal studies. As for the PT-SCZ association, although it was validated by several additional analyses such as SMR-HEIDI and reverse MR, a single SNP-driven MR result is by nature suspect due to the unexplored pleiotropy [16, 28]. Because the volume reduction of PT has been demonstrated in high-risk psychosis and first-episode SCZ patients [29], we suggest that the inferred causality between PT and SCZ is plausible.

There were some limitations to this study. Firstly, classic MR methods largely depend on high heritability and strong exposure instruments [16, 28]. However, both heritability and number of instruments [17, 18] vary across the tested neuroimaging parameters, such that the power of MR is inconsistent across all DTI-Disorder and RD pairs. In fact, 67 DTI-Disorder and 239 RD pairs were discarded at the beginning of our analysis due to the absence of instruments. Even if there were causal links among them, they would not have been detected in our study. Thus, negative results for DD/RD pairs with limited instruments are not as convincing as positive results for those with adequate instruments. For the positive results, it should also be noticed that only the genetically regulated proportion of polymorphisms are associated with the disorders. Secondly, the original GWAS sample sizes may have impacted the MR results since estimation accuracy (i.e., standard error for effect size) for the instrument–outcome relationship is directly linked to the confidence interval of the MR effect estimate [16, 28]. Since GWAS OCD and TS recruited fewer than 10,000 cases, their MR results were underpowered. In fact, several DD/RD pairs for OCD and TS had a large MR effect, but their wide CI range resulted in non-significant *P* values. Future GWAS with a larger sample size, both for neuroimaging polymorphism and neuropsychiatric disorders, will provide a better chance to improve our understanding in this field.

## Conclusions

In conclusion, our analysis results demonstrate that, in general, WM structures make a more significant contribution to the etiology of neuropsychiatric disorders—especially BP and AN—than brain subregion volumes. SLF.AxD was causally related to AN; marginally significant relationships were also found between BCC.FA and AD and between PT and SCZ.

## Methods

We obtained publicly available GWAS summary statistics for DTI, ROI, and neuropsychiatric without collecting any individual information. Ethics approval was obtained in each of the original studies; therefore, no further ethics approval was needed for the current study.

### Data collection and preprocessing

The genetic instruments for DTI measurements have been previously described [17]. Briefly, the ENIGMA-DTI pipeline [30] was used to analyze UKBB diffusion MRI data for 17,706 European participants and generate 110 DTI parameters—namely, fractional anisotropy (FA), axial diffusivity (AxD), mean diffusivity, mode of anisotropy, and radial diffusivity, of 21 WM tracts as well as their mean values. The genetic instrument for ROI volumes was also obtained from UKBB GWAS [18], which included 19,629 European participants and used the standard OASIS-30 Atropos template for registration and Mindboggle-101 atlas for labeling [31].

We collected GWAS summary statistics from the following neuropsychiatric studies on European cohorts: (1) Alzheimer disease (AD) [32]; (2) Attention-deficit/ hyperactivity disorder (ADHD) [33]; (3) Anorexia Nervosa (AN) [34]; (4) anxiety disorders (Categorical phenotype) [35]; (5) autism spectrum disorder (ASD) [36]; 6)bipolar disorder (BP) [37]; 7) insomnia [38] 8) major depression disorders (MD) [39]; 9) obsessive-compulsory disorder (OCD) [40]; 10) posttraumatic stress disorder (PTSD) [41]; 11) schizophrenia (SCZ) [42]; 12) Tourette disorder (TD) [43] (Fig. 1). For GWAS from Psychiatric Genetic Consortium (PGC), there were few samples from UKB. For AD GWAS, the UKBB participants were not included in the case-control analysis. For other GWAS, we could not quantify the extent of sample overlap due to the lack of individual information. Since all GWAS used in the current study were conducted in European ancestry, we did not further adjust for the impact of population stratification.

For each DTI and ROI measurement, we retained single nucleotide polymorphisms (SNPs) with *P* < 5 × 10^− 8^ as strong instruments for MR; measurements without a strong instrument were discarded. We removed SNPs with linkage disequilibrium (LD) r2 ≥ 0.001 for each measurement using reference LD data from the 1000 Genomes Project [44]. Data harmonization was applied independently for each DTI-disease (DD) and ROI-disease (RD) pair with the TwoSampleMR R package [45]. Since many of the GWAS summary statistics analyzed in this study did not provide allele frequency information, we did not exclude SNPs based on ambiguous strand error. For all binary phenotypes, we log-transformed the odds ratio to generate the β value.

### Power calculation

We calculated the variance in phenotype explained by each instrument by

$$ < mathdollar>{R}^2=\frac{2\ast EAF\ast \left(1- EAF\right)\ast {\beta}^2}{2\ast EAF\ast \left(1- EAF\right)\ast {\beta}^2+2\ast EAF\ast \left(1- EAF\right)\ast N\ast se{\left(\beta \right)}^2} $$

Where EAF was the effect allele frequency, β was the effect size, N was the sample size, and se(β) was the standard error of effect size. The F statistic was then denoted as

$$F=\frac{{R}^{2}*(N-2)}{1-{R}^{2}}$$

R^2^ and F were used to evaluate power for each instrument. For each RD and DTI-Disorder pair, we calculated the overall MR power using mRnd tool [46], assuming OR = 1.3 and type I error = 0.05. The assumption of OR was based on the actual MR effects passing the significance threshold. We took this assumption because there is limited observational estimation of OR that is currently available. MR power and the number of valid instruments for each pair are recorded in Additional files 2 and 3. Details for all instruments are shown in Additional file 1.

### Calculation of MR effects

For DD/RD pairs with at least two instruments, we performed a meta-analysis of each instrument’s MR effect using the inverse variance–weighted (IVW) method. The results were considered preliminary results and were used for downstream analyses. For the top IVW findings, we additionally applied weighted mode [47], weighted median [13], and MR-Egger regression [48] approaches—which are relatively robust against horizontal pleiotropy [15]—to further confirm the validity of the MR effect. For DD/RD pairs with only one instrument, estimates based on the Wald ratio were considered preliminary results. For the top Wald ratio finding, we used summary data-based MR (SMR) [49] to confirm the existence of the MR effect and heterogeneity in dependent instruments (HEIDI) [49] to rule out the probability that the MR effect was driven by colocalization of the instrument with the effective locus.

Since both IVW and Wald ratio results were taken into account, the *p*-value was adjusted by the Bonferroni method by numbers of all DTI-Disorder pairs (1320) or ROI-Disorder pairs (1212).

### Sensitivity analysis

The intercept of the Egger regression was used as an indicator of potential horizontal pleiotropy, while modified Cochran’s Q for IVW and Rucker’s Q for Egger regression [50] were used as an indicator of heterogeneity. We used MR pleiotropy residual sum and outlier (MR-PRESSO) [19] with the number of permutations = 2500 to detect potential outlier SNPs for each DD/RD pair and generate an overall *p*-value for heterogeneity; those with outlier(s) were reanalyzed by IVW after removing the outlier(s). We also applied leave-one-out tests for all top findings to further evaluate the effects of unknown outliers.

For all MR results, we tested the reverse MR effect (i.e., neuropsychiatric disorders as exposure and DTI/ROI as outcome) by selecting SNPs with *P* < 5 × 10^− 8^ for each neuropsychiatric disorder as instruments. The absence of a reverse MR effect (IVW *P* > 0.05) was considered as evidence for the validity of directionality.

### Analysis of the general causal contribution

To assess the general contribution of DTI (ROI) polymorphisms to neuropsychiatric disorder, we pooled the IVW P values for all DTI-Disorder (RD) pairs and compared them to the null uniform distribution using quantile-quantile plots. A positive bias (inflation) from uniform distribution was considered as evidence for a general contribution. The significance of inflation was evaluated with the Kolmogorov–Smirnov (KS) test, while the extent of inflation was assessed with the inflation factor λ, which was calculated by chi-square regression using the GenABEL R package [51]. Since the inflation factor might be overestimated due to the small number of *P* values, we shuffled SNP labels for BP and AN 1,000 times to carry out a permutation test. Permutation *P* < 0.05 was considered evidence for significant inflation. These tests were also separately applied to each disease and repeated after removing heterogeneous results (those with Cochran’s *P* < 0.05) or outlier SNPs (those with MR-PRESSO *P* < 0.05).

## Supplementary Information

**Additional file 1.** Summary statistics for all instrument variables after data harmonization.

**Additional file 2.** Full results for DTI-Disease association analysis.

**Additional file 3.** Full results for ROI-Disease association analysis.

**Additional file 4.** Funnel plots for top MR findings, leave-one-out results for top DTI-Disorder pairs, general contribution of DTI to neuropsychiatric disease after sensitivity test adjustment, Rank-Rank overlaps between MR effect and heterogeneity for ROI-Disease analysis.

## Data Availability

Disease GWAS summary data were downloaded from https://www.med.unc.edu/pgc/download-results/. GWAS summary of neuroimaging data were downloaded from https://github.com/BIG-S2/GWAS.

## Abbreviations

2SMR: 2-sample Mendelian randomization.
AD: Alzheimer Disease
AN: Anorexia Nervosa
BP: Bipolar disorder
DTI: Diffusion tensor imaging
ROI: Region-of-interest
