# Supplementary material for: Mendelian randomization studies of brain MRI yield insights into the pathogenesis of neuropsychiatric disorders
Source: BMC Genomics. 2021 Jun 2;22(Suppl 3):342. doi: 10.1186/s12864-021-07661-8 (PMC8171058; doi:10.1186/s12864-021-07661-8)
Supplement: Supplementary file 4 — Additional file 4. Funnel plots for top MR findings, leave-one-out results for top DTI-Disorder pairs, general contribution of DTI to neuropsychiatric disease after sensitivity test adjustment, Rank-Rank overlaps between MR effect and heterogeneity for ROI-Disease analysis. [file 12864_2021_7661_MOESM4_ESM.docx]

**Funnel plots for top DD pairs.**

**
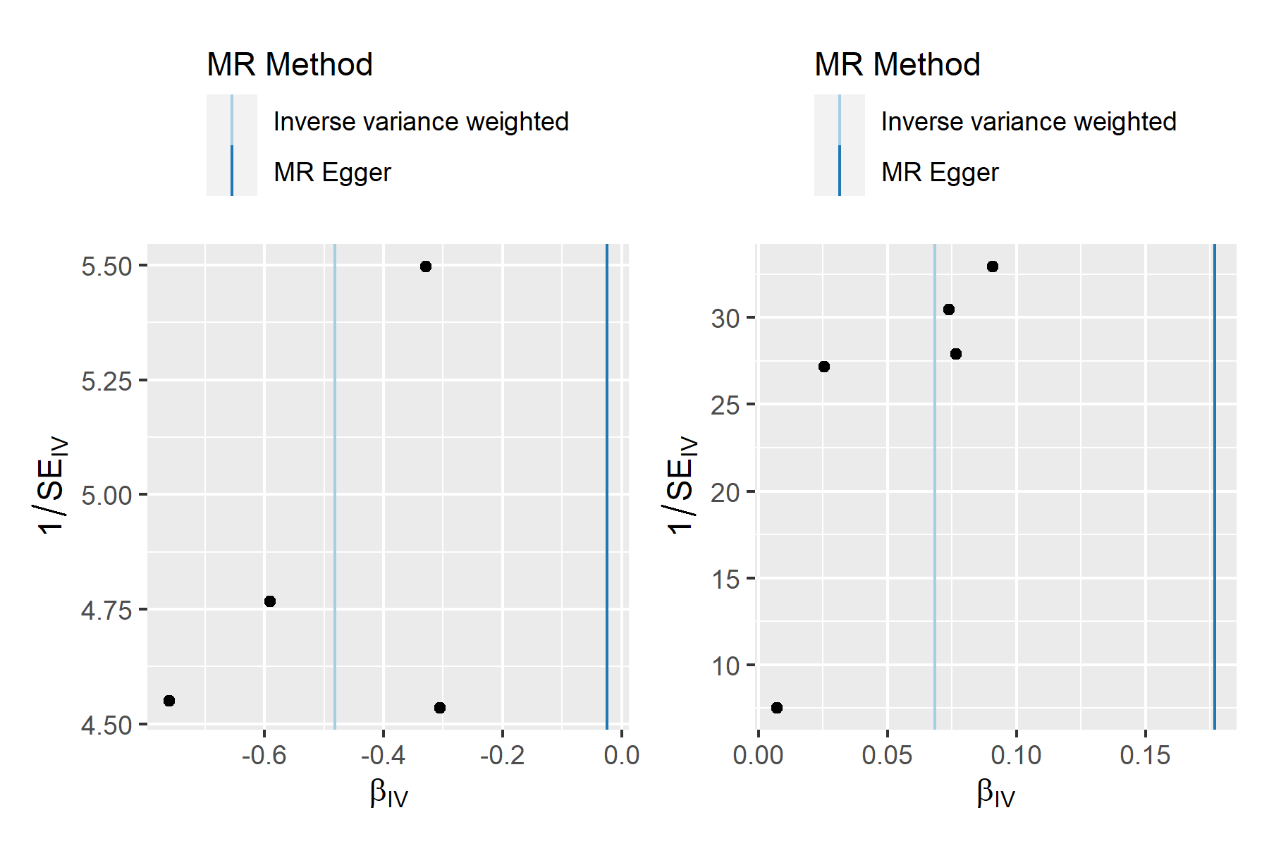
**

x-axis: MR estimation for each SNP; y-axis: inverse Standard Error for MR estimation of each SNP.

**Leave-one-out results for top DD pairs.**

A: SLF.AxD-AN; B: BCC.FC-AD; C: PTR.FA-BP; D: FX.FA-BP; E: CGH.MD-BP; F: RLIC.RD-BP; G: ALIC.FA-ADHD. The order is corresponded to table 1 in main text.

General contribution of DTI to NP disease after sensitivity test adjustment.

**A:** Quantile-Quantile (QQ) plot showing the distribution of all MR p values for the DTI-Disease (DD) association without heterogeneity. **B**: QQ plot showing the distribution of all MR p values after removing outlier SNPs for the DTI-Disease (DD) association. **C:** QQ plot showing the distribution of all MR p values for the DTI-Bipolar Disorder association without heterogeneity. **D**: QQ plot showing the distribution of all MR p values after removing outlier SNPs for the DTI- Bipolar Disorder association.

Rank-Rank overlaps between MR effect and heterogeneity for ROI-Disease analysis.

NS: non-significant. Nominal: p<0.05. SD: Single Disease level significance, p<0.05/110. SW: Study-Wide significance, p<0.05/1253.
